# Deep learning models for deriving optimised measures of fat and muscle mass from MRI

**DOI:** 10.1038/s41598-025-07867-w

**Published:** 2025-07-17

**Authors:** Belvin Thomas, M. Adam Ali, Fatima M. H. Ali, Anthony Chung, Manjiri Joshi, Sophia Maiguma-Wilson, Gabrielle Reiff, Hadil Said, Pardis Zalmay, Michael Berks, Matthew D. Blackledge, James P. B. O’Connor

**Affiliations:** 1 Division of Radiotherapy and Imaging, The Institute of Cancer Research, London, UK; 2 Radiology Department, St George’s University Hospitals NHS Foundation Trust, London, UK; 3 Radiology Department, Northwick Park Hospital, Harrow, UK; 4 Division of Cancer Sciences, University of Manchester, Manchester, UK; 5 Radiology Department, The Christie NHS Foundation Trust, Manchester, UK

**Keywords:** Biomarker, Cachexia, MRI, Oncology, Sarcopaenia, Wellbeing, Prognostic markers, Cancer imaging, Outcomes research, Magnetic resonance imaging

## Abstract

Fat and muscle mass are potential biomarkers of wellbeing and disease in oncology, but clinical measurement methods vary considerably. Here we evaluate the accuracy, precision and ability to track change for multiple deep learning (DL) models that quantify fat and muscle mass from abdominal MRI. Specifically, subcutaneous fat (SF), intra-abdominal fat (VF), external muscle (EM) and psoas muscle (PM) were evaluated using 15 convolutional neural network (CNN)-based and 4 transformer-based deep learning model architectures. There was negligible difference in the accuracy of human observers and all deep learning models in delineating SF or EM. Both of these tissues had excellent repeatability of their delineation. VF was measured most accurately by the human observers, then by CNN-based models, which outperformed transformer-based models. In distinction, PM delineation accuracy and repeatability was poor for all assessments. Repeatability limits of agreement determined when changes measured in individual patients were due to real change rather than test-retest variation. In summary, DL model accuracy and precision of delineating fat and muscle volumes varies between CNN-based and transformer-based models, between different tissues and in some cases with gender. These factors should be considered when investigators deploy deep learning methods to estimate biomarkers of fat and muscle mass.

## Introduction

In oncology, cachexia with fat depletion^1^, obesity with excess intra-abdominal visceral fat^2^ and muscle loss (termed sarcopaenia)^3^ are all well-documented indicators of poor prognosis in patients with cancer^4^. Body fat percentage can be estimated by densitometry methods or bioelectrical impedance analysis which are low cost and simple to use, but not widely available^5^. Muscle mass can be assessed using anthropometry measures including body-mass index (BMI), skin-fold thickness, and body circumference (e.g., waist, thigh, and calf), which are simple and readily available in any clinical setting but considered inaccurate^6,7^.

Medical imaging offers an alternate, accurate and reliable way to measure the spatial distribution and volumes of fat mass and muscle mass, such that CT and MRI are currently considered as gold standard methods in body composition analysis^8^. Fat mass can be assessed by measuring subcutaneous fat (SF) as a surrogate for total body fat (TF). In part this is due to the relative ease of determining these measurements^9^but measuring intra-abdominal visceral fat (VF) may have a greater prognostic relevance to overall wellbeing than SF, particularly in male subjects^4^. Muscle mass is often measured using the psoas muscle (PM) as a surrogate for total skeletal muscle (TM), since the psoas is a readily recognisable anatomical landmark when measured at the L3 level. However, these assessments are often limited to one or a few slices of CT data and substantially under-sample the external muscle (EM) mass visualised with CT or MRI.

Deep learning (DL) models^10,11^ can be used to extract quantitative biomarkers from clinical images. These models, typically follow an encoder-decoder structure. During training, the encoder extracts semantic representations from input images (e.g. CT or MRI) and the decoder learns to produce more precise outputs from them. They eliminate the need for hand-crafted features designed or implemented by human observers and can learn to make predictions directly from data by integrating multi-scale features from images^12,13^.

The U-Net configuration^13,14^ is the most widely used benchmark in medical image segmentation tasks due to its flexibility and numerous successful applications in biomedicine. It combines low-level and high-level features via skip connections between encoder and decoder. Originally, U-Net was formulated with a Convolutional Neural Network (CNN) based encoder and decoder^14^. Since then, various architectural modifications^15–17^ were proposed, and nnU-Net^18^ made this fine-tuning process relatively obsolete by providing a standardised baseline framework for CNN-based implementations. In recent years, transformer-based networks have been applied at various stages of the classic encoder-decoder structure owing to their ability to capture long-range dependencies and global context^19,20^.

Quantitative imaging biomarkers—such as those quantifying fat and muscle—can directly inform patient well-being and guide treatment decisions. However, multiple technical, biological and clinical validation steps must be performed to translate biomarkers from discovery phase into effective tools that alter patient management^21^.

Here, we use DL models to estimate volumes of muscle and fat derived from T_2_-weighted (T_2_W) abdominal MRI images. We compare the accuracy and repeatability of estimates of tissue volumes to understand variations across the DL models, including assessment of the effect of gender. Finally, we demonstrate how repeatability information can be used to inform how fat and muscle mass change during an intervention, in this case treatment with chemotherapy.

## Results

### Multi-class segmentation of abdominal tissue volumes

**Fig. 1:**
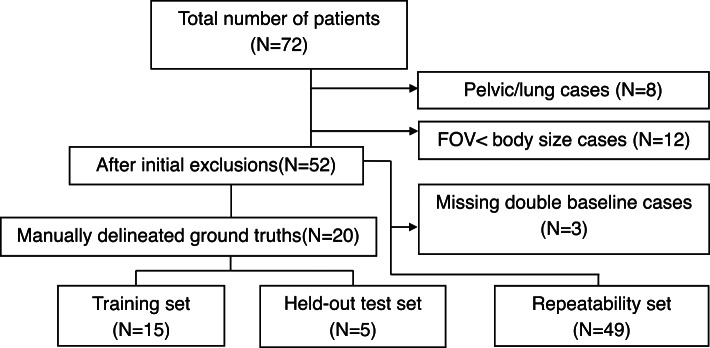
Flow diagram showing the selection of abdominal volumes satisfying double baseline requirement for DL model training and evaluation.

72 patients were evaluated in the study, but *N* = 8 did not have imaging centred on the abdomen. A further *N* = 12 had a field of view (FOV) that was less than the craniocaudal body size and had noticeable wrapping artefacts. Of the 52 patients with usable data, *N* = 3 patients had only one baseline MRI scan. The final dataset comprised of 49 patients (summarised in Fig. 1).

**Fig. 2:**
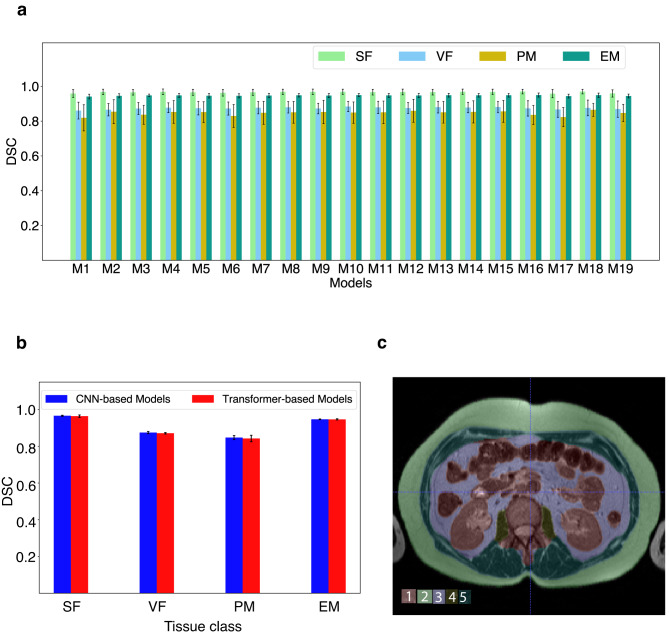
Model accuracy in held-out test patients. (**a**) Plot of DSC of the four tissue target volumes (SF, VF, PM and EM; abbreviations defined in the text) across 19 models. (**b**) Mean DSC of tissue volumes for groups of CNN-based (M1–M15) and Transformer-based (M16–M19) models after combining the data in panel (**a**). (**c**) Sample segmented mid-abdominal slice from a test volume with respective class labels.

Each tissue class in held-out test volumes (*N* = 5; using all 19 DL models) had high Dice Similarity Coefficient (DSC) values and showed little variation (0.96 ≤ SF ≤ 0.97; 0.86 ≤ VF ≤ 0.88; 0.82 ≤ PM ≤ 0.87; 0.94 ≤ EM ≤ 0.95). This indicated strong segmentation performance of the models across the classes (Fig. 2a).

Across all 19 models, SF and EM performed slightly better than VF and PM. No significant variation in performance was seen between CNN-based and Transformer based model outputs across the four tissue classes (Fig. 2b). Representative segmented slice from a test volume with respective class labels based on inferences from Model M3 is shown (Fig. 2c).

### Comparison of DL model accuracy against human observers

**Fig. 3:**
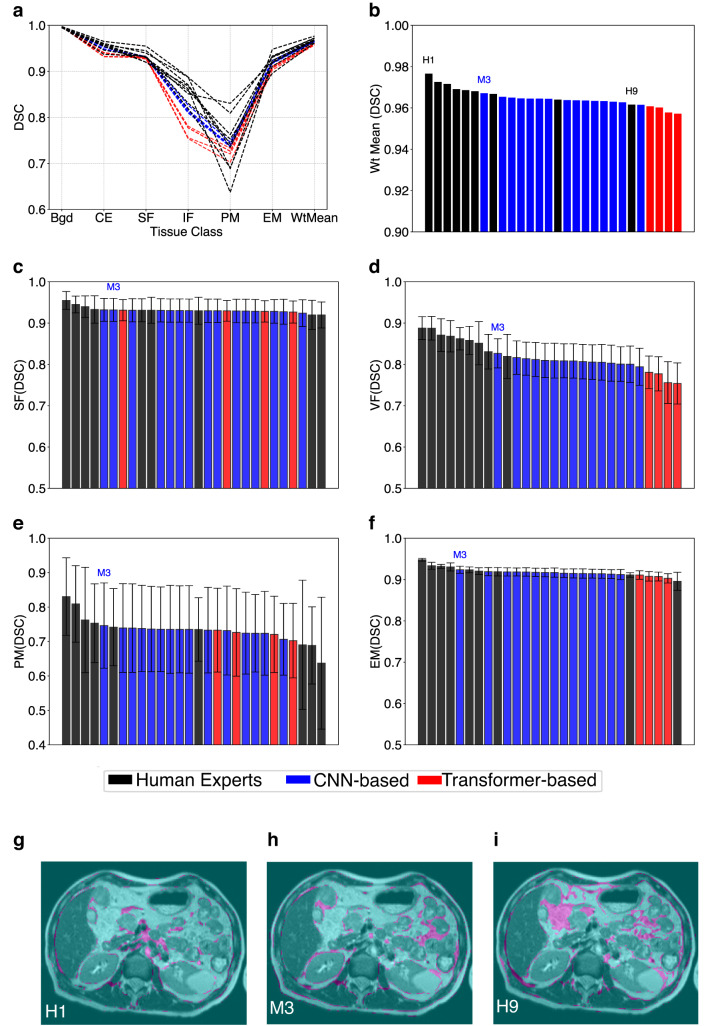
Comparison of human experts versus DL models. (**a**) Class-wise DSC (based on STAPLE stack) and weighted mean for 9 annotators and 9 DL Models (just the best 5 CNN models are selected for visual clarity). (**b**) Distribution of weighted average DSC across all 9 manual and 19 auto segmentations. Distribution of DSC for each of the four tissue classes—(**c**) SF, (**d**) VF, (**e**) PM, (**f**) EM—are shown in descending order of performance. In each case, the position of model M3 is indicated. Finally, inter-observer variations are shown in pink and compare (**g**) best human rater H1, (**h**) best performing model M3, and (**i**) worst human rater H9 against the STAPLE ground truth.

The DL models were compared against annotations from 9 human observers with STAPLE-generated consensus segmentation as the ground truth. Performance of both human observers and DL models was relatively poor in VF and PM classes (Fig. 3a). In all other tissue classes, models and annotators performed well and there were only marginal differences between them. In general, CNN-based models performed slightly better than transformer-based models.

The weighted average performance of some of the CNN-based models come closer to the best-performing human observers (Fig. 3b). Viewing each tissue class separately showed that negligible difference in performance for delineating background (as expected for this simple task) or cavity excluded tissue (Supplementary Fig. 1).

Negligible difference in delineation accuracy was seen for SF or EM with DSC of 0.931 ± 0.007 (range 0.929 to 0.955) and 0.917 ± 0.010 (range 0.896 to 0.948) respectively. However, there was markedly worse delineation accuracy seen for VF with DSC of 0.818 ± 0.035 (range 0.754 to 0.888) and PM with DSC of 0.733 ± 0.034 (range 0.637 to 0.831).

Human observers generally performed best for SF, VF and EM. However, CNN models outperformed human observers for PM, where the worst 3 performances were all by humans (Fig. 3c–f). Representative analysis of inter-observer variations on T_2_W images in Fig. 3g–i shows variations(pink-shaded areas) of best human annotator (H1), best performing DL Model (M3) and worst human annotator (H9) against the STAPLE-generated reference. The VF was frequently difficult to delineate, as highlighted in all three example images.

Analysis of another overlap-based metric (Intersection over union; IOU) and a boundary-based metric (Normalised Surface Dice; NSD) also confirmed that M3 was the best performing DL model (Supplementary Figs. 2–3). Analysis of a second boundary-based metric Hausdorff Distance 95 (HD95)—that calculates the 95th percentile of distances between the predicted and ground truth boundaries—showed M3 to perform best for segmentations aside from VF or PM (Supplementary Fig. 4).

**Fig. 4:**
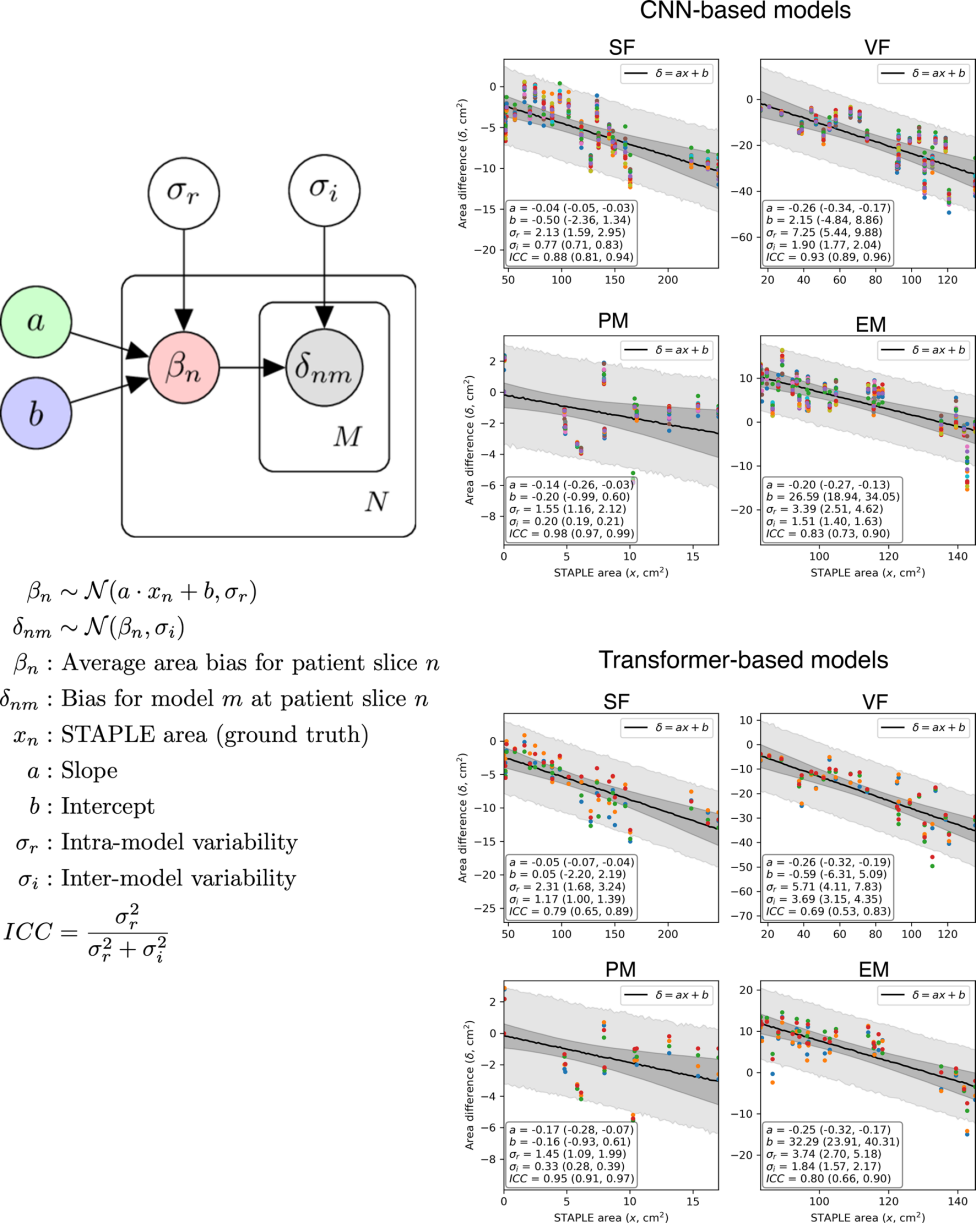
Bias and inter-/intra-model variability. Left: Our hierarchical, linear model of the difference between STAPLE ground truth and AI-predicted area for each patient slice (*N* = 25) in our reader study. Right: Results from fitting our model to CNN-based and transformer-based AI models independently. Estimated parameters are demonstrated in the lower-left quadrant of each figure, with 95% confidence intervals (from HMC sampling) shown in parentheses. Coloured symbols represent the difference for each of the AI models (different colour per model), the darker shaded area represents the 95% confidence in the linear fit, whilst the lighter shaded area represents the 95% posterior predictive distribution for *δ*_nm_.

Our hierarchical Bayesian model revealed a significant linear trend in the bias of estimated area across the range of STAPLE-estimated areas for all anatomical regions (see Fig. 4). At the mean STAPLE area, the bias was estimated to be -4.4%, -22.9%, -18.7%, and + 5.2% for SF, VF, PM and EM respectively for CNN-based models. For transformer-based models, this was − 5.3%, −26.6%, − 20.6%, and + 5.5%. The intraclass correlation coefficient (ICC) was found to be significantly higher than 0.5 in all cases, indicating that the inter-model variability in AI segmentations is relatively low compared to the intra-model variations and thus models perform similarly to one another. In all parameters we achieved $\:\widehat{r}$ = 1.00, indicating excellent performance^22^of our HMC sampler.

### Gender-based comparison of tissue volumes

Since males and females are known to have differences in the amount and distribution of fat and muscle, we evaluated the effect of gender on volumes of each tissue class. In the baseline scans (*N* = 49), volumes of each tissue class were computed using all 19 DL models and averaged to present a gender-based comparison.

**Fig. 5:**
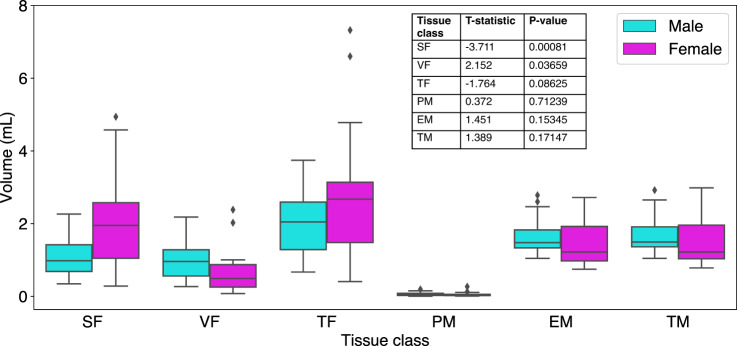
Gender-based volume comparison of tissue classes. Volumes of each tissue class from baseline scans (*N* = 49) are computed using all 19 DL models and averaged to present a gender-based comparison. SF and VF differ by gender as expected (SF is higher in female whereas VF is higher in males), and the differences are shown to be statistically significant (p-value < 0.05).

Significant differences of tissue class volume (*p* < 0.05) were found for SF (higher volumes in females) and VF (higher volumes in males) (Fig. 5). No differences were seen when the two fat tissue compartments were combined into TF, since these differences counterbalanced one another. No differences were seen for tissue class volumes of muscle.

### Repeatability assessment of DL models

**Fig. 6:**
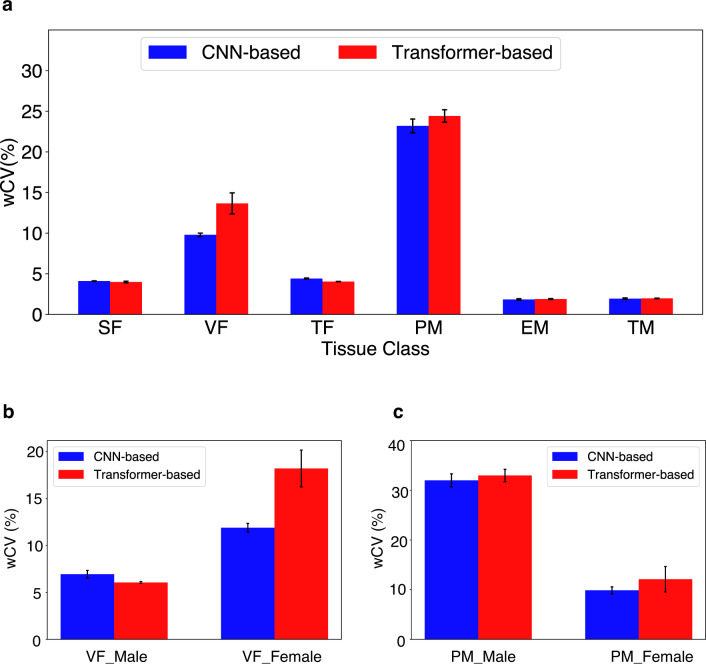
Assessment of DL model precision across different model architectures (M1-M15 CNN-based models versus M16-M19 transformer-based models) for each tissue classes. (**a**) Repeatability metric wCV is used across the entire patient population (*N* = 49) to show that repeatability varies between tissue classes. There is no significant difference between CNN-based and transformer-based models except for VF. Gender-based differences in repeatability are seen in (**b**) where VF is less repeatable in females than males, accounting for the difference between CNN-based and transformer-based models. (**c**) PM is more repeatable in females, but there are no differences seen between CNN-based and transformer-based models.

The test–retest precision of DL models has seldom been assessed, since this requires double baseline imaging in the absence of treatment. Here, we found that repeatability varies between tissue classes (Fig. 6a). Calculating the mean, standard deviation and range of within-subject Coefficient of Variation (wCV) for the 19 models (Supplementary Fig. 5) showed that SF wCV (4.10 ± 0.03% with range 4.06 to 4.15 for CNN-based and 3.98 ± 0.10% with range 3.89 to 4.08 for Transformer-based) was more repeatable than VF wCV (9.79 ± 0.21% with range 9.24 to 10.12 for CNN-based and 13.65 ± 1.30% with range 12.44 to 14.83 for Transformer-based), the latter of which also showed variable repeatability between type of DL model used with CNN-based models more repeatable than Transformer-based models. The TF wCV was between that seen in the two individual fat tissue classes. For muscle, EM wCV (1.83 ± 0.09% with range 1.63 to 1.92 for CNN-based and 1.89 ± 0.05% with range 1.83 to 1.95 for Transformer-based) was much lower than that of the PM wCV (23.20 ± 0.84% with range 22.16 to 25.03 for CNN-based and 24.42 ± 0.76% with range 23.31 to 24.94 for transformer-based), with greater than 10-fold difference. TM was similar to EM since the latter formed the vast majority of tissue contributing to TM, relative to PM.

Since we found differences in volumes of tissue by gender, we examined whether this influenced repeatability of tissue volumes. VF was less repeatable in females than males (Fig. 6b). The values in females drove the relatively high wCV seen for all subject VF, as well as the difference in performance by CNN-based and transformer-based models. There were also some gender differences that were not apparent when viewing all subject data; the relatively high wCV seen for all subject PM was heavily driven by male subjects irrespective of DL model, with female subject PM wCV being within the range seen for other tissue volumes with best performing repeatability (Fig. 6c). No gender effect was seen for SF or EM (Supplementary Fig. 6).

For comparison, Intra-class Correlation Coefficient (ICC) were calculated for each DL model. All values were high, typically exceeding 0.9. Consequently, there were negligible differences seen by gender (Supplementary Fig. 7).

### Longitudinal assessment of changes in patients

**Fig. 7:**
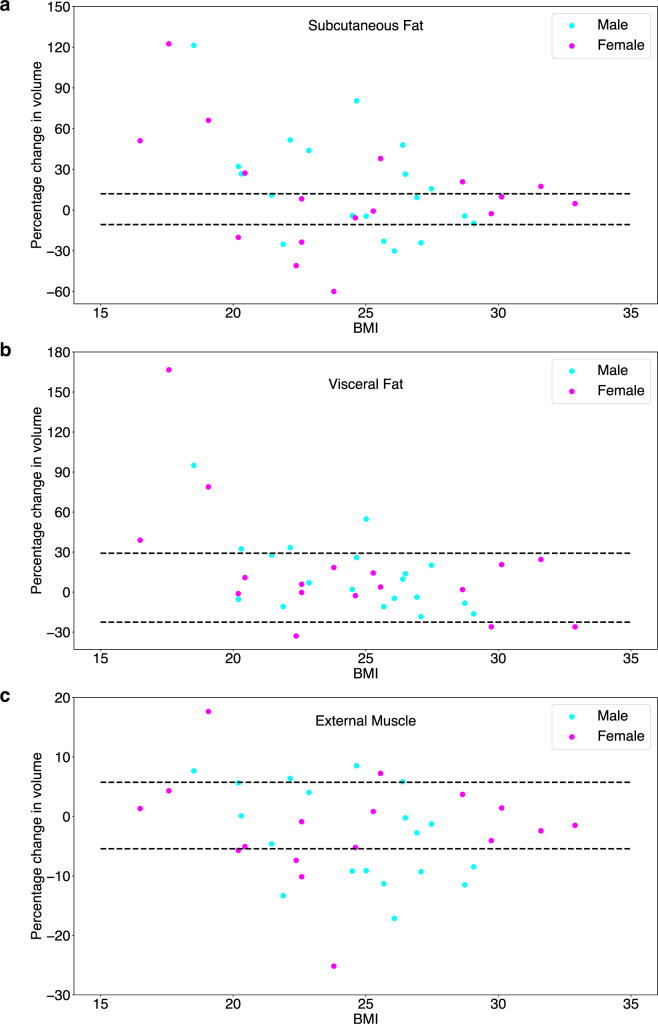
Longitudinal assessment of changes in patients. Plots of percentage changes in volumes of (**a**) SF, (**b**) VF and (**c**) EM. The changes from baseline images to 180 days on treatment are coloured based on gender and are shown across various BMI categories, for those patients with data at both pretreatment and at 180 days. Individual changes (coloured dots) are compared to the percentage LOA for each tissue class (dotted lines).

Asymmetric RC Limits Of Agreement (LOA) were computed for each DL model (Supplementary Table ST1) and showed minimal differences. Since M3 (LowRes 3D U-Net) performed consistently as well or better than other DL models, this was selected for assessing longitudinal changes of fat and muscle in patients. LOA percentages for M3 for each tissue volume (decrease followed by increase) were − 10.7 and 12.0 for SF, − 22.6 and 29.1 for VF and − 5.2 and 5.5 for EM. Percentage changes in tissue volumes of SF, VF and EM were evaluated from baseline images to 180 days on treatment and compared to the percentage LOA for each tissue class (Fig. 7a–c).

16 patients (42.1%) had an increase in SF and 8 patients (21.1%) had a decrease in SF by 180 days. Only 6 patients (15.8%) had an increase in VF and 3 patients (7.9%) had a decrease in VF by 180 days, with the majority (76.3%) having no detectable change with a 95% confidence. 5 patients (13.2%) had an increase in EM and 12 patients (31.6%) had a decrease in EM by 180 days. In general, there was no discernible difference in changes according to gender. In distinction, no tissue class showed evidence of cohort change over the 180-day period (all *p* > 0.5, see Supplementary Table ST2).

## Discussion

Artificial intelligence algorithms, particularly those applying DL models are being used increasingly in medical research. To translate these methods into clinical practice, such models must undergo validation.

In this study, we evaluated DL in the context of multi-class segmentation, which is a non-trivial problem especially when different compartments are of widely varying size/volume. Compared to normal population, this problem needs more attention in patients with debilitating diseases like cancer where body characteristics change quickly over time. More specifically, this necessitates an added requirement of ensuring repeatability of methods developed in this space. Here, we show how DL models vary in their accuracy and repeatability of defining volumes of fat and muscle volumes, depending on the precise anatomical class of tissue. This is likely because tissue boundaries vary in ease of definition and in size.

The SF is bound externally by the background outside of the patient and internally by the EM outer margin. The EM is bound internally by the VF outer margin. These three margins are relatively simple to delineate. Consequently, the SF and EM were shown to have accuracy similar to that of human observers (DSC ≥ 0.9) and both tissue classes were highly repeatable irrespective of choice of DL model with wCV of around 2–4%. These features make volumes of SF and EM potentially good biomarkers of fat and muscle mass respectively, from a technical point of view.

In distinction, some tissue classes have margins that are difficult to define. PM has a small volume relative to the three other tissue classes examined and DL models had an accuracy measured by DSC of around 0.7 to 0.75. Interestingly, human observers variably performed better or worse than DL models, but the best performance was only slightly better than DSC of 0.8. PM was also markedly less repeatable as a tissue class, with wCV of around 25%. While 1D or 2D measurements of psoas on single slice CT images remains a popular surrogate for muscle mass, our data suggest that volume of PM a relatively poor choice for a biomarker of muscle mass on MRI, from a technical point of view. If tissue segmentations based on machine learning are to be deployed, then our data support measuring the entire muscle within a field of view and not the PM alone.

The VF performed somewhere between these two extremes. DSC measurement of accuracy was around 0.8 in CNN-based models, noticeably better than in transformer-based models, but worse than seen in 8/9 human observers. VF repeatability was around 10% with the better performing CNN-based models, but analysing the data split by gender showed that in males—who had larger volumes of VF—the precision estimate was around 5%, indistinguishable from that of SF and EM.

Biomarker selection should take account of biological, clinical and technical factors as well as cost effectiveness^21^. Distributions of body fat vary between men and women and these differences may have prognostic significance. Therefore, while volume of VF measured by DL from T_2_W MRI may perform slightly less well than volume of SF, the two may have different biological or clinical value (distinct from their technical performance).

We then demonstrated a potential application for using precision measurements to evaluate individual patients on therapy. Cohort statistics did not detect any changes in the tissue volumes of fat or muscle. However, analysis of percentage changes compared to the RC LOA for each tissue class revealed that around half of all patients had significant change in their volume of SF and EM. This discrepant change—with both increases and decreases seen in individuals in both tissue classes explained the lack of cohort level change. This finding may reflect a combination of patients who were initially cachectic then gaining fat and muscle bulk, as well as patients who were not cachectic then gaining excess mass due to treatment effects or ill health. In distinction, the majority of patients had unchanged volumes of VF.

The choice of model for defining DL-derived biomarkers is also important. Overall, CNN-based models performed equally or better than transformer-based models in this study, with most noticeable differences seen when delineating VF and PM volumes, with the M3 model performing best in this study. nnU-Net framework aims to maximise the potential of original U-Net architecture using a set of standardised preprocessing steps and carefully designed training strategies. This low-code solution reduces the chances of making mistakes in the otherwise error-prone process where individual developers must take decisions related to tuning of various training parameters that could negatively affect model predictions down the line.

Nevertheless, the superiority of CNN-based model configurations has been challenged by some transformer-based configurations recently, where the encoder networks are designed following the transformer architecture^23–25^. While CNN-based models and transformer-based models compete against each other for superiority in multiclass segmentation problems^26–30^, in this study we provide evidence that CNN-based models can outperform transformer models in the circumstances examined here.

There were no significant differences observed in the under-/over-segmentation of CNN versus transformer-based models, as derived from our Bayesian analysis of delineated areas from our multi-reader study. We could therefore conclude that both methods performed similarly well in this context. However, it is important to note that generally larger delineated regions tended to be more biased than smaller regions, as might be expected. Interestingly, the variation in delineated area between different models was small in comparison to the intra-model (patient-based) variation, again indicating that models performed similarly well when deriving area from delineated regions.

Some study limitations exist. The numbers of patients examined (*N* = 49) was modest, but the data was rich in that it included test-retest scans, enabling examination of biomarker repeatability. This assessment of precision is seldom undertaken in evaluation of DL model performance. Since data was MRI, the entire abdomen was not included, neither was any other body location. Care was taken to place the image field of view in the same anatomical location, but inevitably this will cause some sampling error. Model performance of measuring tissue volumes should be robust across data acquired at different field strengths, sequence protocols, and vendors, but further multi-centre and multi-vendor validation is required to confirm these data and to ensure findings are not due to over-fitting. Variations in protocol were partially addressed with this study since some patients had 8 mm slice thickness in their T_2_W volume acquisition, compared to the majority who had 4 mm slice thickness. Also, evaluation of healthy volunteer data may provide a useful control, to ensure that changes measured in this study were treatment related.

Nonetheless, the fact that repeatability wCV was around 5% for several tissue class volumes indicates that error due to malpositioning of image field of view was likely minimal in this single centre study with a harmonised acquisition protocol. Also, the performance of transformer-based models can be influenced by various parameters beyond those examined in this study, such as the number of attention heads and learning schedule. Further research is required to systematically investigate the impact of these factors.

In conclusion, we have performed assessments of the accuracy and precision of DL model estimates of the volumes of different fat and muscle tissue classes, to examine their technical value as potential biomarkers. In this dataset CNN-based U-Net models performed well and support further translation as biomarkers in health and disease.

## Methods

### Patient population and dataset

We performed retrospective evaluation of T_2_W abdominal MRI images from colorectal cancer patients acquired in a clinical trial^31^. Ethical approval was granted from the Central Manchester Research Ethics Committee (REC Reference 09/H1008/99) and the study was registered with EudraCT (number 2009-011377-33). All methods were performed in accordance with these regulations.

All patients gave written informed consent, were aged ≥ 18 years, had primary metastatic colorectal cancer, and had a performance status of 0–2. Patients received bevacizumab 10 mg/kg for one cycle of therapy, followed by combined oxaliplatin 85 mg/m^2^, folinic acid 175 mg, and bevacizumab 5 mg/kg were administered as short infusions along with a bolus 5FU 400 mg/m^2^ was administered and followed by 5FU 2400 mg/m^2^ over 46 h. This was administered on a 2-weekly schedule.

MRI acquisition and analysis was performed to Good Clinical Practice standards. Data were acquired on a 1.5 T Philips Achieva scanner (Philips Healthcare, Best, The Netherlands). Abdominal imaging was performed where patients had lesions of ≥ 3 cm in the liver. Patients were intended to have two pre-treatment baseline scans followed by post-treatment scans performed at 180 days. Multi-slice 2D T_2_W turbo spin-echo images were acquired (FA = 90°, TR = 541 ms, TE = 80 ms, with field of view (FOV) = 375 mm × 264 mm, acquired in-plane resolution = 1.46 mm × 1.84 mm, reconstructed in-plane resolution = 1.46 mm × 1.46 mm, 25 axial slices, either 4 or 8 mm thick). Imaging was performed without breath-holds or gating. All images (*N* = 134) extracted from the dataset were converted from Analyze 7.5 to NIFTI format.

### Defining ground truth

Ground truth volumes for training (*N* = 15) and testing (*N* = 5) the DL models were defined by a standardised manual delineation process as shown in Supplementary Fig. 8. A subset of scans was selected randomly from different patients (*N* = 20) covering all visits, so that one scan per patient was chosen. Six target volumes were labelled as 0:Background (Bgd), 1:Part of abdominal cavity excluded from study (Cavity-excluded), 2:SF, 3:VF 4:PM and 5:EM. The manual annotation was performed using ITK-SNAP platform. In addition, the TF was computed from addition of SF and VF, and the total muscle TM was computed from addition of PM and EM.

### Training of DL models for multi-class segmentation of abdominal tissue volumes

A collection of CNN-based and transformer-based models were developed and trained. CNN-based models were trained by leveraging the nnU-Net V2 framework^18^ while the transformer-based models were trained using UNETR^24^ and Swin-UNETR^23^ architectures (terminology for all DL steps is defined in supplementary material). All four configurations of U-Net (2D, 3D full-resolution, 3D low-resolution and 3D cascade; distinctions explained in supplementary material) were implemented using nnU-Net framework, were trained with five-fold cross validation and were ensembled. Similarly, the models from five-folds trained using both UNETR and Swin-UNETR networks were also ensembled. Each fold was trained using a split of 12 volumes for training and 3 volumes for validation. During training we adhered to the default parameters in the original implementations unless an improvement in performance was observed, to make a fair comparison between the performance of different architectures.

All models were developed and trained on a dedicated workstation hosting Dual NVIDIA Quadro RTX 8000 GPUs. Data augmentations and hyperparameters in CNN-based models were determined automatically as part of the self-configuration of nnU-Net pipeline. In the transformer-based models, we compared the outcome from multiple experiments to determine the appropriate combinations. In each fold, the training was allowed to continue until the mean validation Dice score performance^32^ plateaued, and the best-performing model was selected for testing. Pre-processing scripts, model training routines and testing scripts were written using MONAI 1.1 ecosystem in a Python 3.10 environment running PyTorch 1.3. Since nnU-Net V2 is now integrated into the MONAI framework we used this integrated version for training the CNN-based models. At various stages of development, the outputs were visually inspected using ITK-SNAP 4.0.1.

Training CNN-based models: nnU-Net models were trained over 150 epochs with 250 iterations per epoch. Validation mean-Dice scores computed at the end of each epoch were used to keep track of the training progress. All models used Stochastic Gradient Descent (with Nesterov momentum = 0.99) as optimiser with an initial learning rate of 0.0001 and DiceCEloss^33^ as the loss function. Other specific details related to the four different U-Net configurations are detailed in supplementary material (see supplementary table ST3 and related notes).

Training Transformer-based models: The transformer-based models were trained for 37,500 iterations and validation mean dice scores computed at the end of every 250 iterations were used to keep track of the training progress. The PyTorch- based training routine accepted 3D image patches selected randomly in batches of four from each of the 12 training volumes and produced a six-channel output with each channel representing the probability of a voxel to belong to one of the six individual classes. All models used AdamW as optimiser with an initial learning rate of 0.0001 and DiceCEloss^33^ as the loss function.

The preprocessing pipeline employed various techniques to standardise input data and enhance model generalisation. Input images were normalised to a standardised range (0.0–1.0), without clipping outliers and thereby ensuring consistent inputs to the network while preserving relative intensity relationships within the images. All volumes were resampled to a uniform voxel spacing using bilinear interpolation for images and nearest-neighbour interpolation for segmentation masks, preserving both intensity gradients and precise label boundaries. Additionally, all volumes were reoriented to a consistent Right-Anterior-Superior (RAS) anatomical orientation, eliminating potential inconsistencies in spatial representation. Patch-based sampling strategy and automatic foreground extraction were introduced to balance the proportion of foreground and background voxels in training samples. This reduced unnecessary computation on background regions while focusing the effort on anatomically relevant areas.

To enhance the training set and prevent overfitting, we included multiple data augmentation approaches in the pipeline. Geometric transformations such as random flipping along each spatial axis (with 10% probability per axis) and random rotation of 90, 180 and 270 degrees (with 10% probability) were used. These were complemented by intensity augmentations in the form of random brightness shifts of magnitude ± 0.10 applied with 50% probability, helping the model become robust to variations in image acquisition parameters.

During training, we experimented by varying the size of input 3D blocks and decoder network features in the MONAI-based implementation^34^ and selected the final model composition based on better validation mean dice scores. In the final model composition, UNETR folds accepted 3D image patches of size 16 × 96 × 96 at a normalised spatial resolution of 4 mm x 1.5 mm x 1.5 mm and the decoder network feature size was 16. swin-UNETR folds accepted patches of size 96 × 96 × 96 at a normalised spatial resolution of 1 mm x 1.5 mm x 1.5 mm and the decoder network feature size was 36. More details on these experiments and other model configuration parameters in the final implementation are given in the supplementary material.

Formation of ensembles: At the end of training, ensembles were formed by selecting the best models from each fold. U-Net variants (2D U-Net, 3D U-Net (fullres, lowres, cascade)) were ensembled via averaging to create models M1–M4. These models were further combined in pairs (M5–M10), triples (M11–M14), and a full ensemble (M15) to form all possible combinations in the nnU-Net framework. Additionally, models from five-fold training using UNETR and Swin-UNETR were ensembled using averaging (M16, M18) and voting (M17, M19) methods. Table 1 summarises the definition of different model configurations.

**Table 1:** Configuration of deep learning model ensembles.

Model number	Configuration	Model number	Configuration
M1	2D U-Net	M11	Ensemble(M1, M2, M3)
M2	3D U-Net (full resolution)	M12	Ensemble(M1, M2, M4)
M3	3D U-Net (low resolution)	M13	Ensemble (M1, M3, M4)
M4	3D Cascade	M14	Ensemble (M2, M3, M4)
M5	Ensemble (M1, M2)	M15	Ensemble (M1, M2, M3, M4)
M6	Ensemble (M1, M3)	M16	UNETR (Ensemble by averaging)
M7	Ensemble (M1, M4)	M17	UNETR (Ensemble by voting)
M8	Ensemble (M2, M3)	M18	Swin-UNETR (Ensemble by averaging)
M9	Ensemble (M2, M4)	M19	Swin-UNETR (Ensemble by voting)
M10	Ensemble (M3, M4)		

The inferences computed from all 19 models were post-processed using connected component analysis to keep only the largest connected component. This step ensured that any labels mistakenly drawn outside of abdominal ROI (for example, parts of the arms) were excluded from the final segmented volumes.

### Testing of DL models

The performance of all trained models was rigorously assessed by conducting multiple tests on the segmentation outputs from each DL model. Different metrics were selected to assess model accuracy, inter-observer variations and precision, both with and without gender separation. The performance variations at the level of model architectures were also studied by comparing the metrics of CNN-based and Transformer-based models.

DL model accuracy using held-out test data: As an initial test of segmentation accuracy, the overlap performance of each of the segmented tissue volumes was assessed for all 19 models using DSC which is an overlap-based metric that measures the overlap between predicted regions and actual regions. A subset of five held-out test patients—manually delineated using the same protocol (Supplementary Fig. 8)—served as the reference dataset for computing DSC. Segmentation outputs from each model was used to get a class-wise DSC. Then the final metric was computed by averaging across the test subjects.

DL model comparison of accuracy using human observers: In a further test of segmentation accuracy, nine clinical radiologist experts were recruited to generate ground truths by independent delineation. Each of them manually annotated a test stack of 25 slices, working on ITK-SNAP labelling platform using the protocol described in Supplementary Fig. 8. The test stack was generated by compiling five equispaced slices each from scans of five unique patients whose scans were not used in model training. The labels were then combined using STAPLE^35^ algorithm to generate a consensus stack. Performance of individual annotators and DL models was compared against STAPLE-generated ground truth using class-wise DSC and weighted average.

To be comprehensive, we also performed an equivalent analysis using (a) another overlap-based metric and (b) two boundary-based metrics. IOU is an overlap-based metric that measures the ratio of the intersection of the predicted and ground truth regions to their union. The range of IOU is 0 to 1 (where higher is better). IOU is related to DSC by: IOU = DSC/(2-DSC). Hausdorff Distance 95 calculates the 95th percentile of distances between the predicted and ground truth boundaries to mitigate the impact of outliers (unit: millimetres, where lower values are better). The NSD evaluates how much of the predicted surface is within a certain tolerance distance from the ground truth surface. It normalizes the DSC based on surface distances and has a range of 0 to 1 (where higher is better).

Finally, we explored a hierarchical Bayesian model (Fig. 4) to describe both the bias and inter-/intra-model variability of AI methods for deriving estimates of the area of each segmented region, comparing the AI-derived area with the STAPLE-derived area as ground truth. Upon visualizing these differences, we observed that the bias tended to be linearly dependent on the STAPLE estimate, and thus a linear fit was included in our model. Data from CNN-based and Transformer-based models were fit independently. Posterior distributions of model parameters based on data we derived using Hamiltonian Monte Carlo (HMC) sampling, achieved using the Stan^36^ software package, with the following parameters: number of chains = 3, number of samples per chain = 1500, number of warmup samples = 500, thinning = 1 (none). To ensure that convergence was achieved in all parameters, we calculated the Gelman-Rubin^22^ statistic ($\:\widehat{r}$ ) and deemed good convergence where $\:\widehat{r}$ < 1.1.

Repeatability assessment of DL models: Volume of body compartments is not expected to change significantly for the pair of scans acquired before the start of treatment, but volume measurements are subject to error due to day-to-day variation. Test-retest measurement from two pre-treatment scans was used to assess the model precision for each target volume, following approaches more commonly applied to in MRI measurements of tumours^37^. We used the paired class-wise tissue volumes extracted from all patients (*N* = 49) to evaluate test-retest repeatability across the DL models. Three commonly used metrics—within-subject Coefficient of Variation (wCV), Intra-class Correlation Coefficient (ICC) and asymmetric Limits Of Agreement (LOA)^38^—were evaluated.

### Using preferred DL model for longitudinal assessment of changes in patients

The best performing DL model was chosen based on accuracy and repeatability results. Tissue volumes were computed for each patient for all three MRI visits and a change-trend analysis was performed comparing these segmented volumes at pretreatment and 180 days after start of therapy at the cohort level using paired t-tests. Percentage changes in tissue volumes following therapy were computed in comparison with the average volumes from the two pre-treatment visits and compared with pretreatment body mass index (BMI) measurements. Then, the RC thresholds derived from repeatability assessment were used to define significant tissue volume changes over time. The tissue volume changes exceeding RC thresholds are considered significant with 95% confidence^38,39^.

## Supplementary Information

Supplementary Information.

## Data Availability

Data used in this study are available from the corresponding author on reasonable request. The source code with all the necessary scripts to reproduce data pre-processing, model training, model inference and post-processing operations are shared on GitHub (https://github.com/btho733/ICR-MR).
