# Supplementary material for: Deep learning models for deriving optimised measures of fat and muscle mass from MRI
Source: Sci Rep. 2025 Jul 17;15:25960. doi: 10.1038/s41598-025-07867-w (PMC12271309; doi:10.1038/s41598-025-07867-w)
Supplement: Supplementary file 1 — Supplementary Information. [file 41598_2025_7867_MOESM1_ESM.pdf]

Supplementary Material: Deep Learning models for deriving optimised measures of fat and muscle mass from MRI

Belvin Thomas[1*], M Adam Ali[1, 2], Fatima M H Ali[3], Anthony Chung[2], Manjiri Joshi[2], Sophia Maiguma-Wilson[2], Gabrielle Reiff[2], Hadil Said[2], Pardis Zalmay[2], Michael Berks[4], Matthew D Blackledge[1], James P B O'Connor[1,4,5*]

[1]Division of Radiotherapy and Imaging, The Institute of Cancer Research, London, UK

[2]Radiology Department, St George's University Hospitals NHS Foundation Trust, London, UK

[3]Radiology Department, Northwick Park Hospital, Harrow, UK

[4]Division of Cancer Sciences, University of Manchester, Manchester, UK

[5]Radiology Department, The Christie NHS Foundation Trust, Manchester, UK

***Corresponding authors email addresses:**

belvin.thomas@icr.ac.uk

james.oconnor@icr.ac.uk

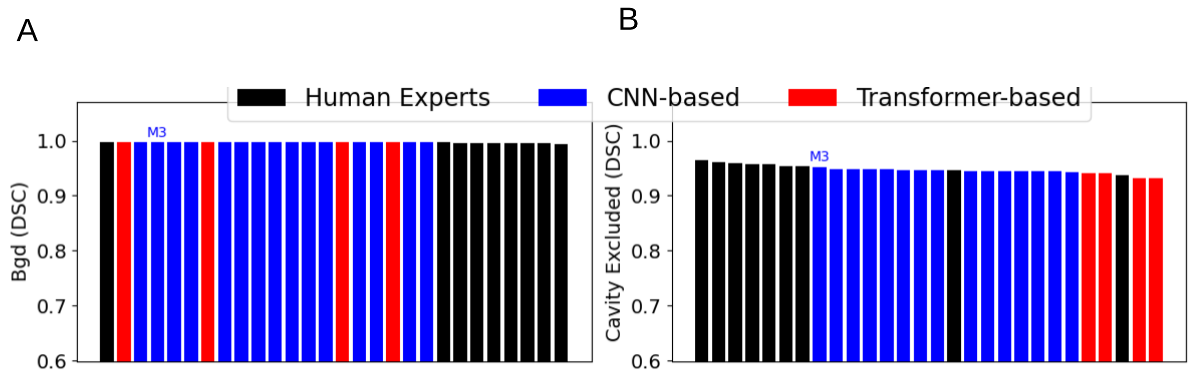

Supplementary Figure 1: Distribution of DSC across all 9 manual and 19 auto segmentations for the classes (A) Background (B) Parts of abdominal cavity excluded from the study.

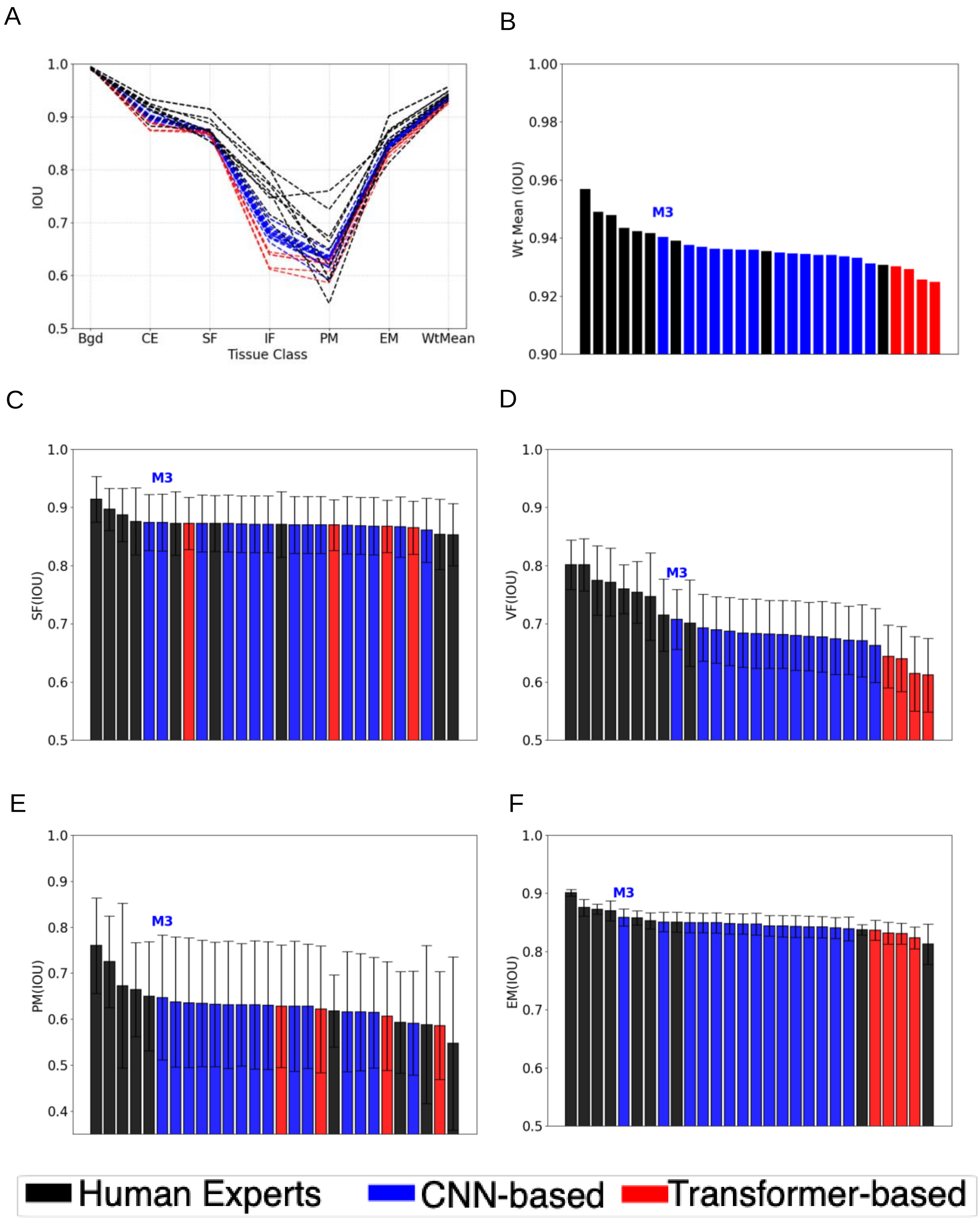

Supplementary Figure 2: Comparison of Human experts versus DL Models using the metric Intersection Over Union (IOU). (A) Class-wise IOU (based on STAPLE stack) and weighted mean for 9 annotators and 19 DL Models. (B) Distribution of weighted average IOU across all 9 manual and 19 auto segmentations. Distribution of IOU for each of the four tissue classes – (C) SF, (D) VF, (E) PM, (F) EM – are shown in descending order of performance. In each case, the position of model M3 is indicated.

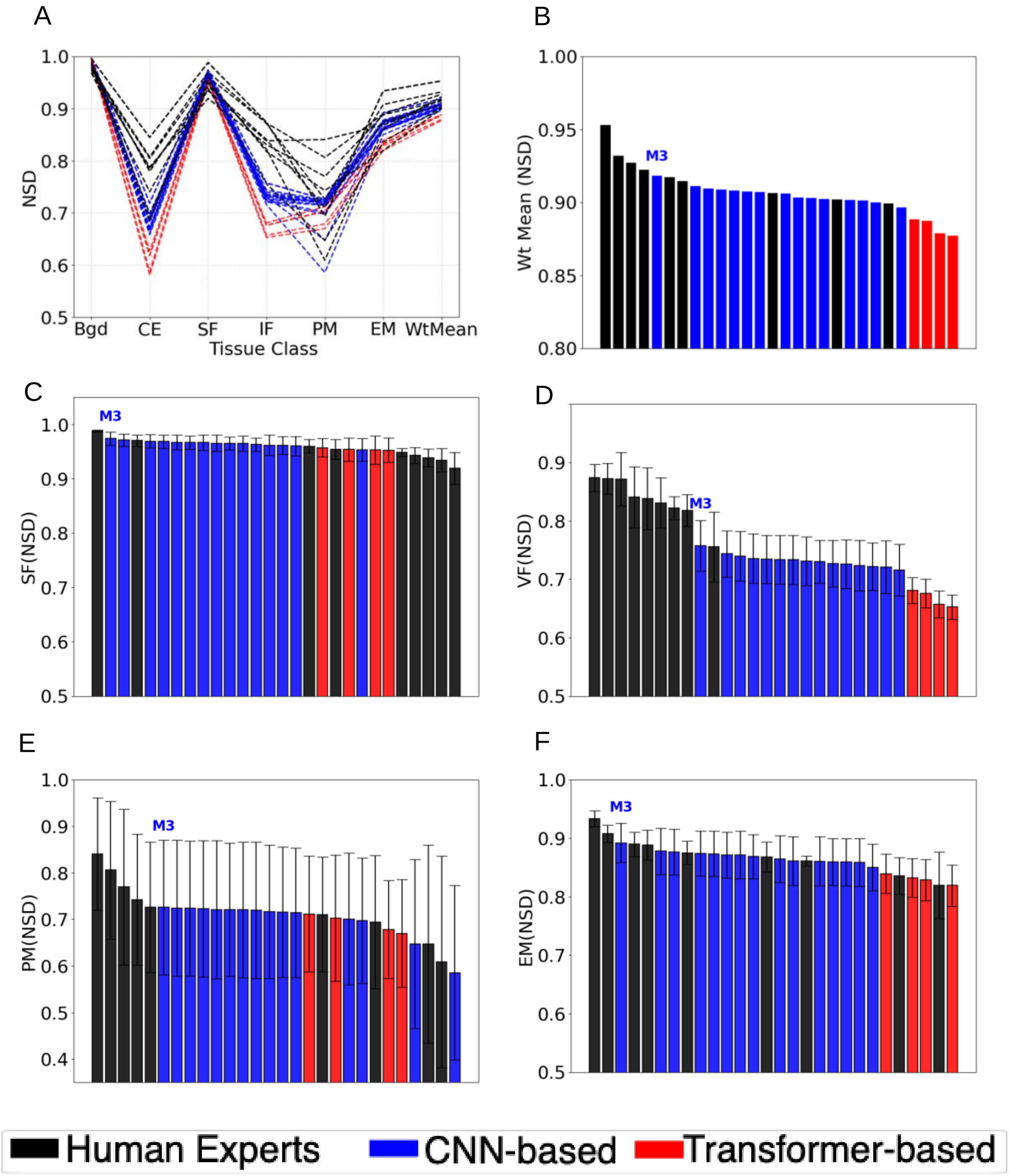

Supplementary Figure 3: Comparison of Human experts versus DL Models using the metric Normalised Surface Dice (NSD). (A) Class-wise NSD (based on STAPLE stack) and weighted mean for 9 annotators and 19 DL Models. (B) Distribution of weighted average NSD across all 9 manual and 19 auto segmentations. Distribution of NSD for each of the four tissue classes – (C) SF, (D) VF, (E) PM, (F) EM – are shown in descending order of performance. In each case, the position of model M3 is indicated.

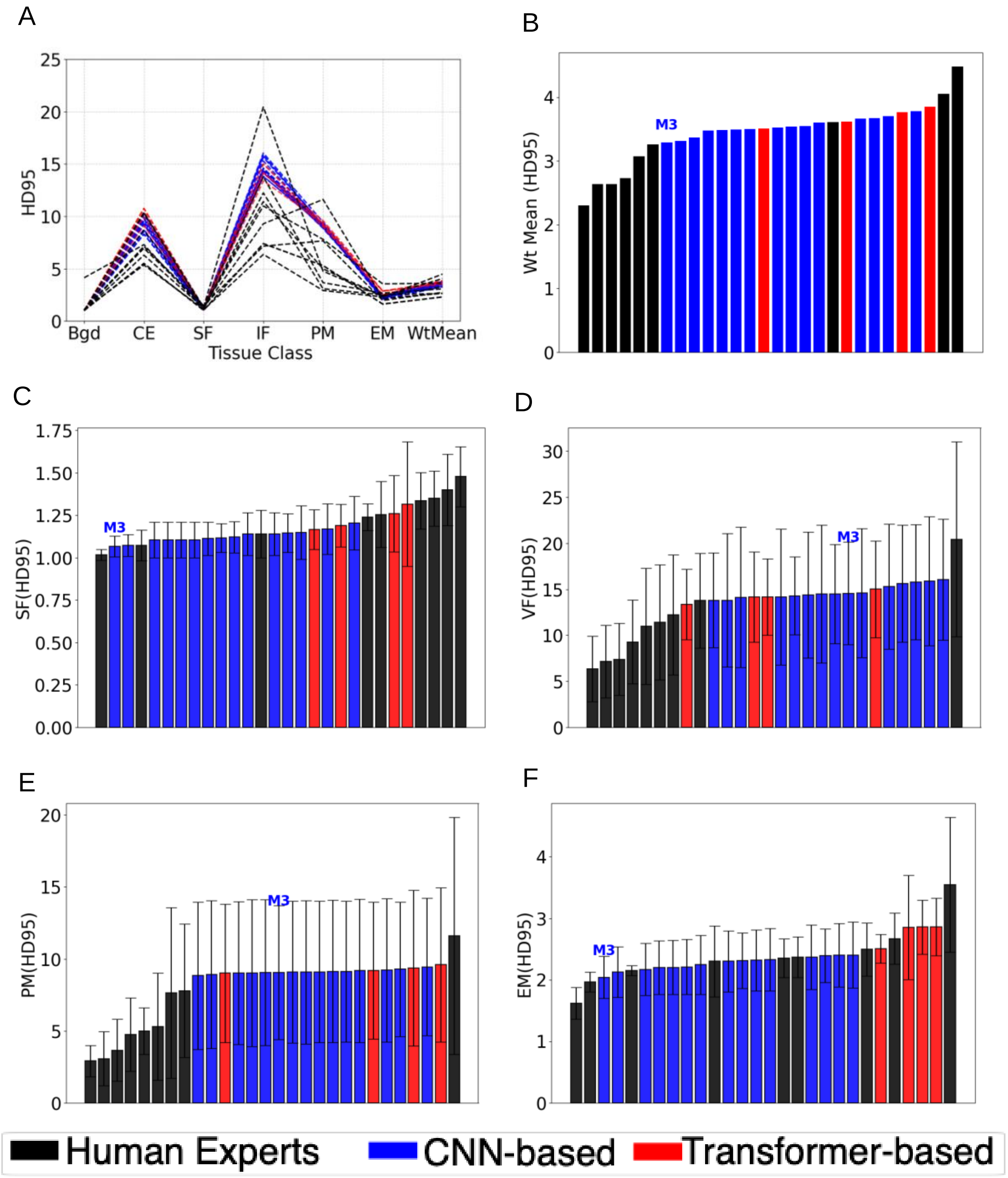

Supplementary Figure 4: Comparison of Human experts versus DL Models using the metric Hausdorff Distance 95th percentile (HD95). (A) Class-wise HD95 (based on STAPLE stack) and weighted mean for 9 annotators and 19 DL Models. (B) Distribution of weighted average HD95 across all 9 manual and 19 auto segmentations. Distribution of HD95 for each of the four tissue classes – (C) SF, (D) VF, (E) PM, (F) EM – are shown in descending order of performance (Lower values are better). In each case, the position of model M3 is indicated.

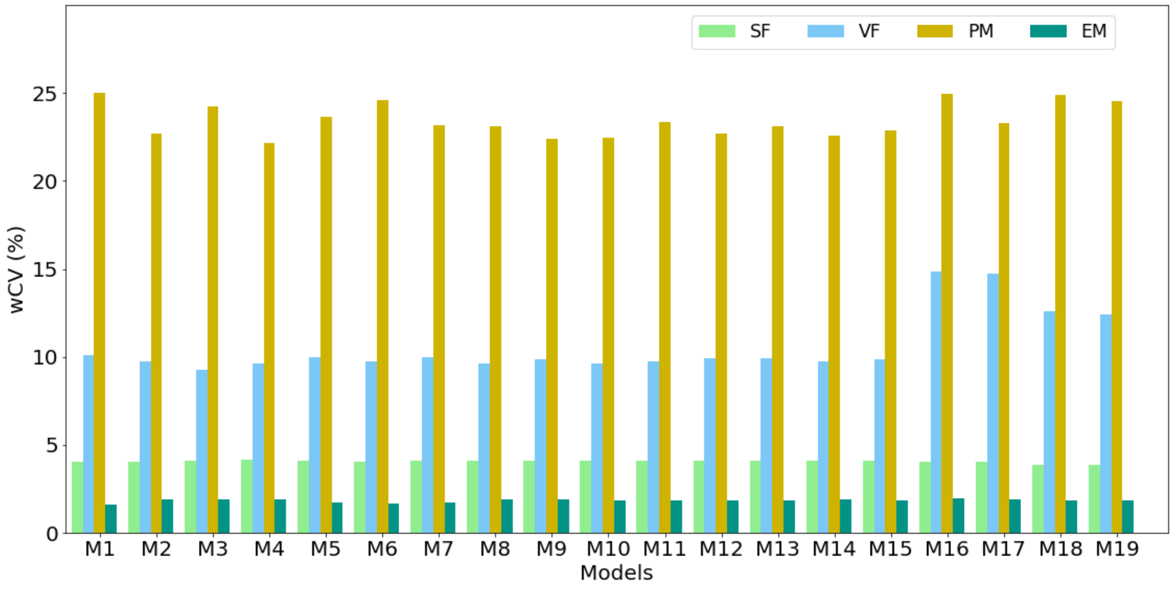

Supplementary Figure 5: Plots of within-subject coefficient of variation (wCV) for each of the tissue volumes – SF,VF,PM and EM - computed across all 19 DL models.

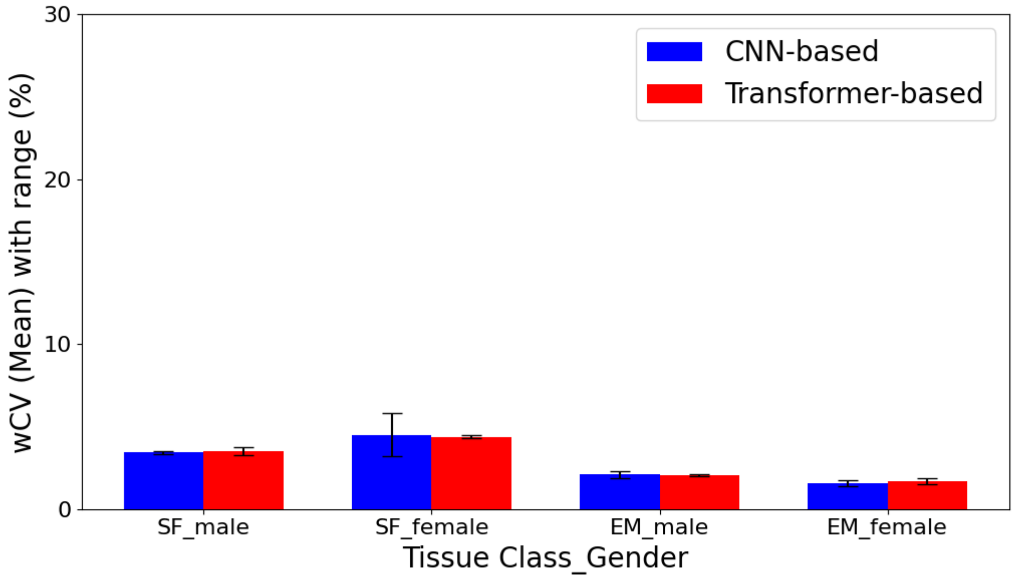

Supplementary Figure 6: Summary of the CNN-based and transformer-based model wCV for SF and EM split by gender.

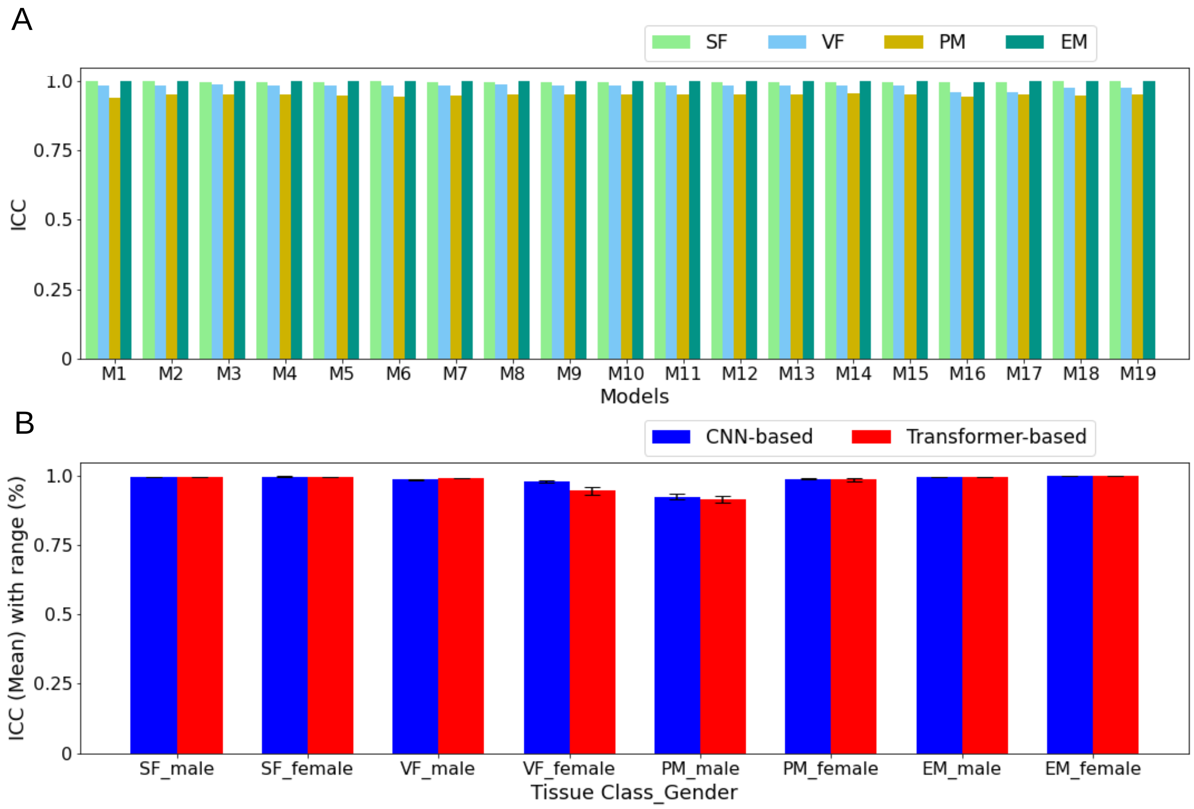

Supplementary Figure 7: Plots of intra-class correlation coefficient (ICC) (A) for each tissue volume computed across all 19 models and (B) with gender considered for each tissue volume.

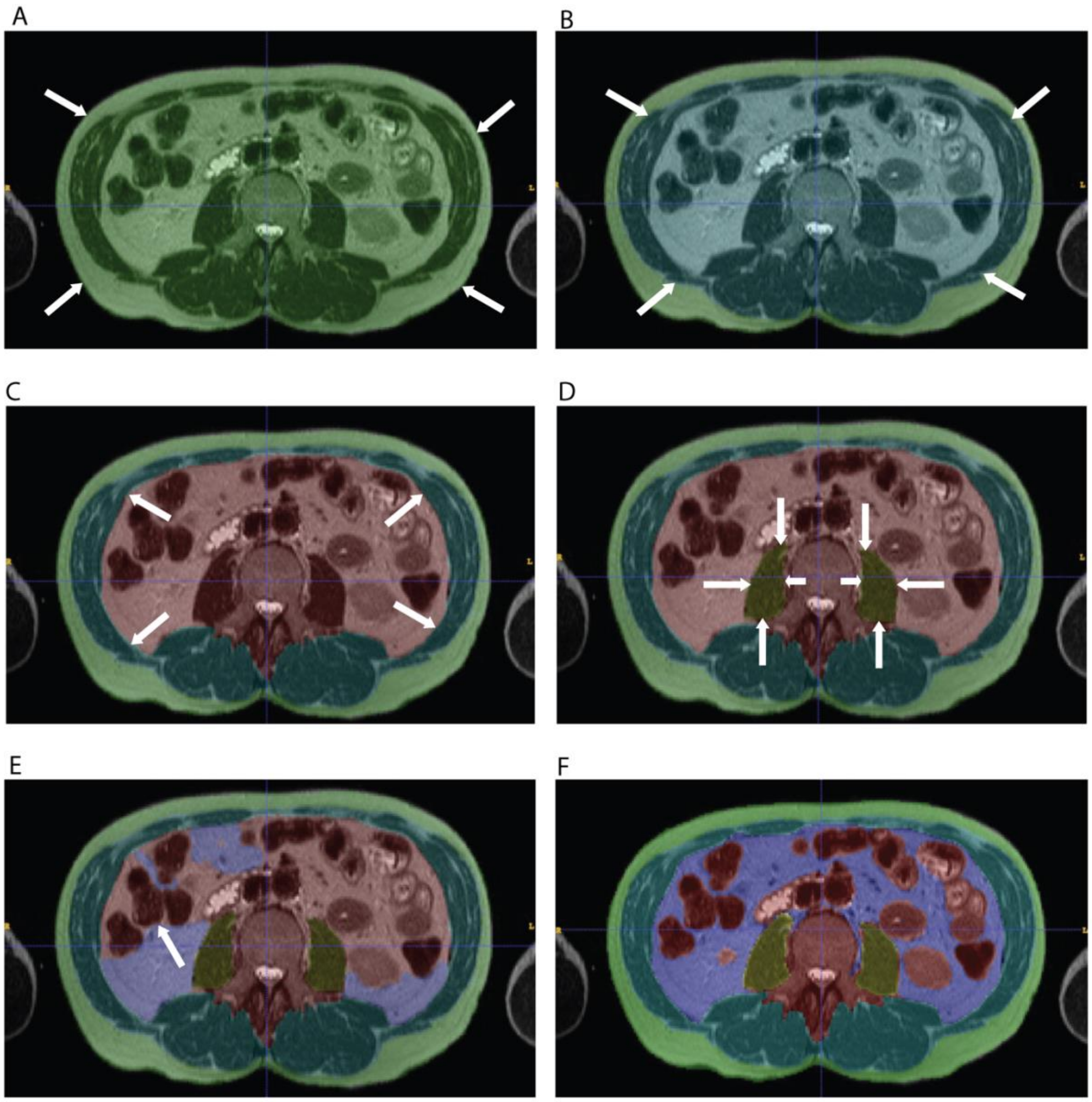

Supplementary Figure 8: Illustrative example showing the steps to manually delineate 6 target volumes in a slice from the T2-weighted images. (A) Outer boundary of body (B) Outer boundary of muscle (C) Outer boundary of the abdominal cavity (D) are defined. Next, the two psoas muscles are delineated in full. Finally, the intraperitoneal fat is drawn round, as shown at (E) an intermediate point and (F) when completed. Combination of these steps produces volumes labelled 0-5 in the text.

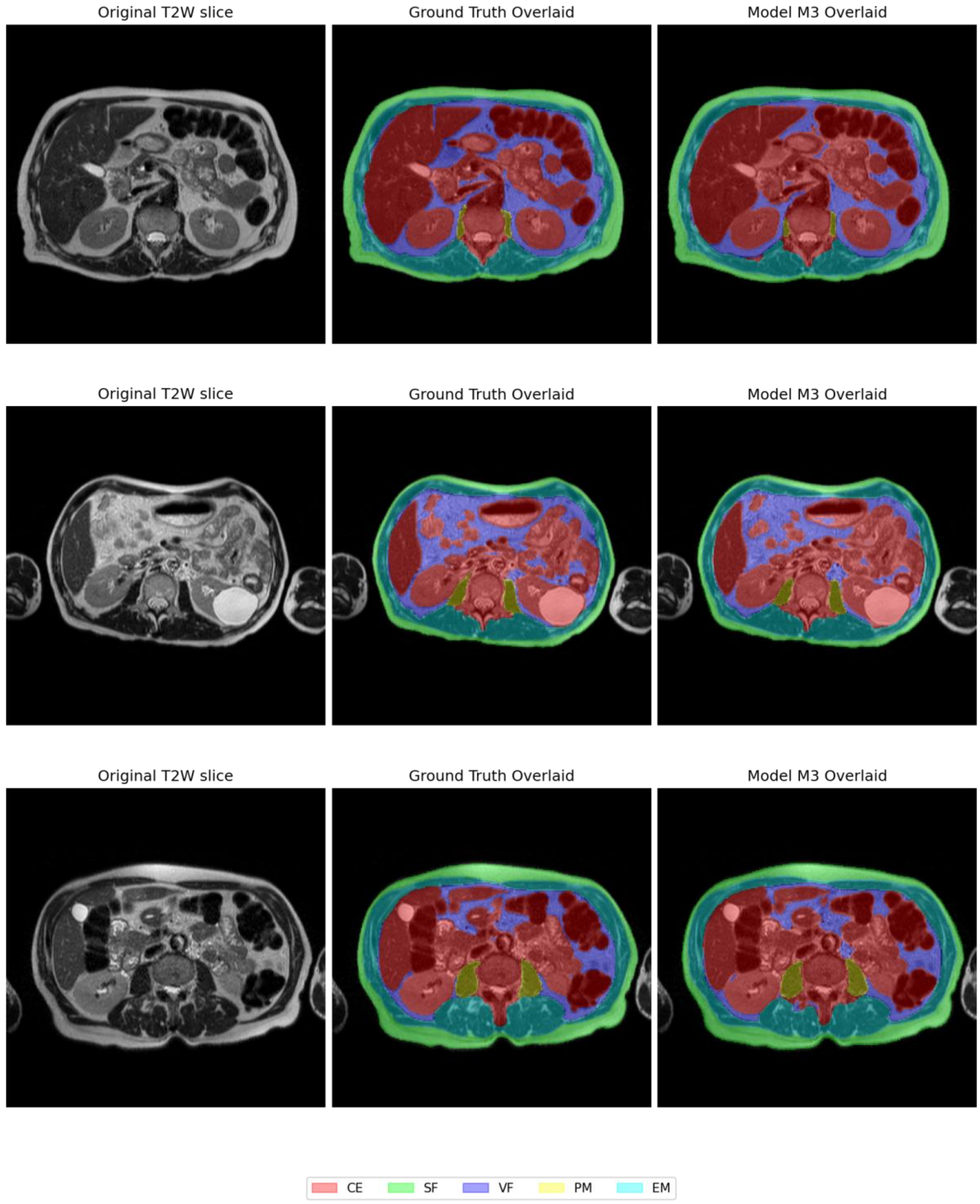

Supplementary Figure 9 : Segmentation results from multiple test patients – sample 1. Each row displays an axial slice from T2W MRI (left), STAPLE-derived ground truth segmentation overlay (middle) and automated segmentation results from the selected DL model M3 (right).

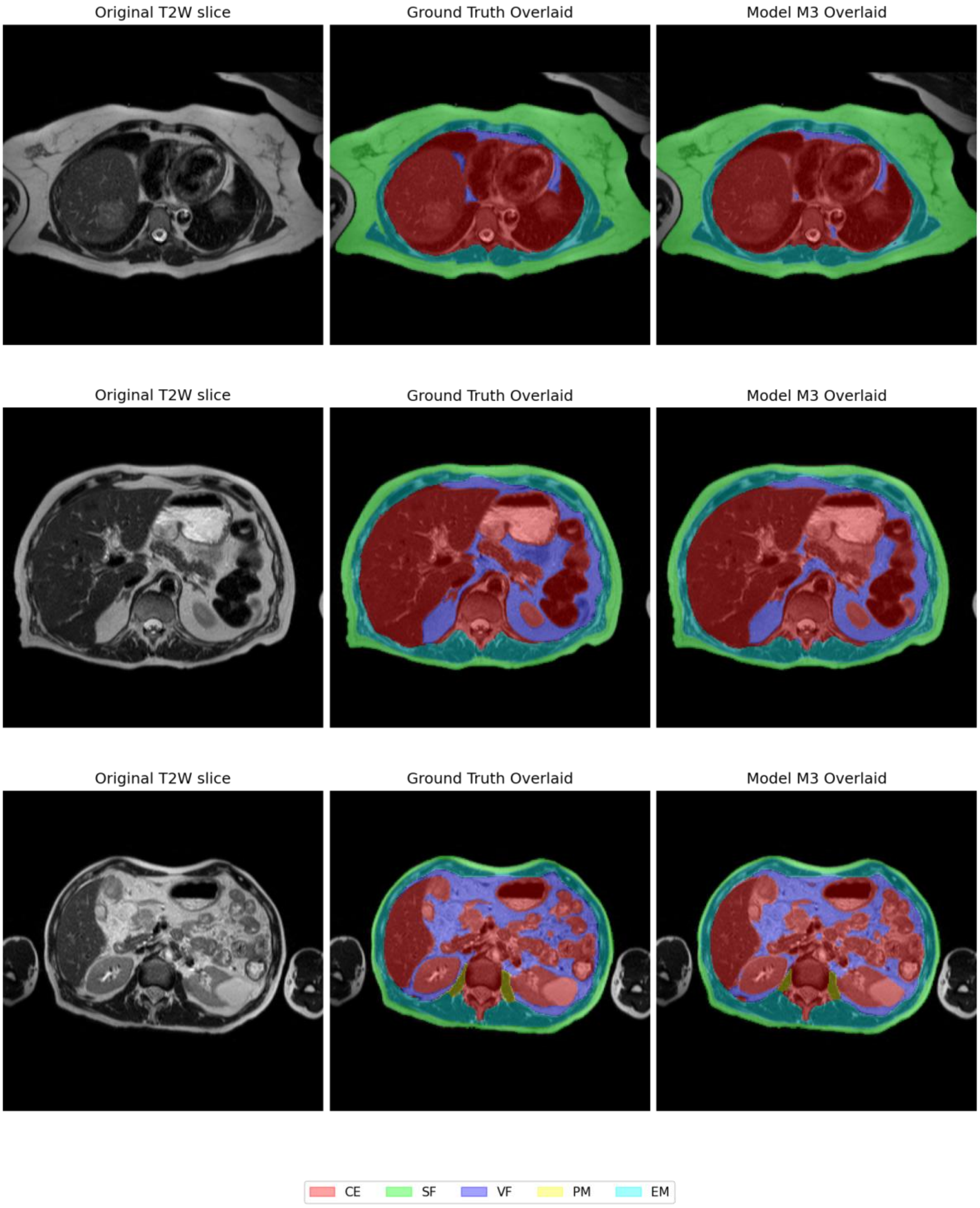

Supplementary Figure 10: Segmentation results from multiple test patients - sample 2. Each row displays an axial slice from T2W MRI (left), STAPLE-derived ground truth segmentation overlay (middle) and automated segmentation results from the selected DL model M3 (right).

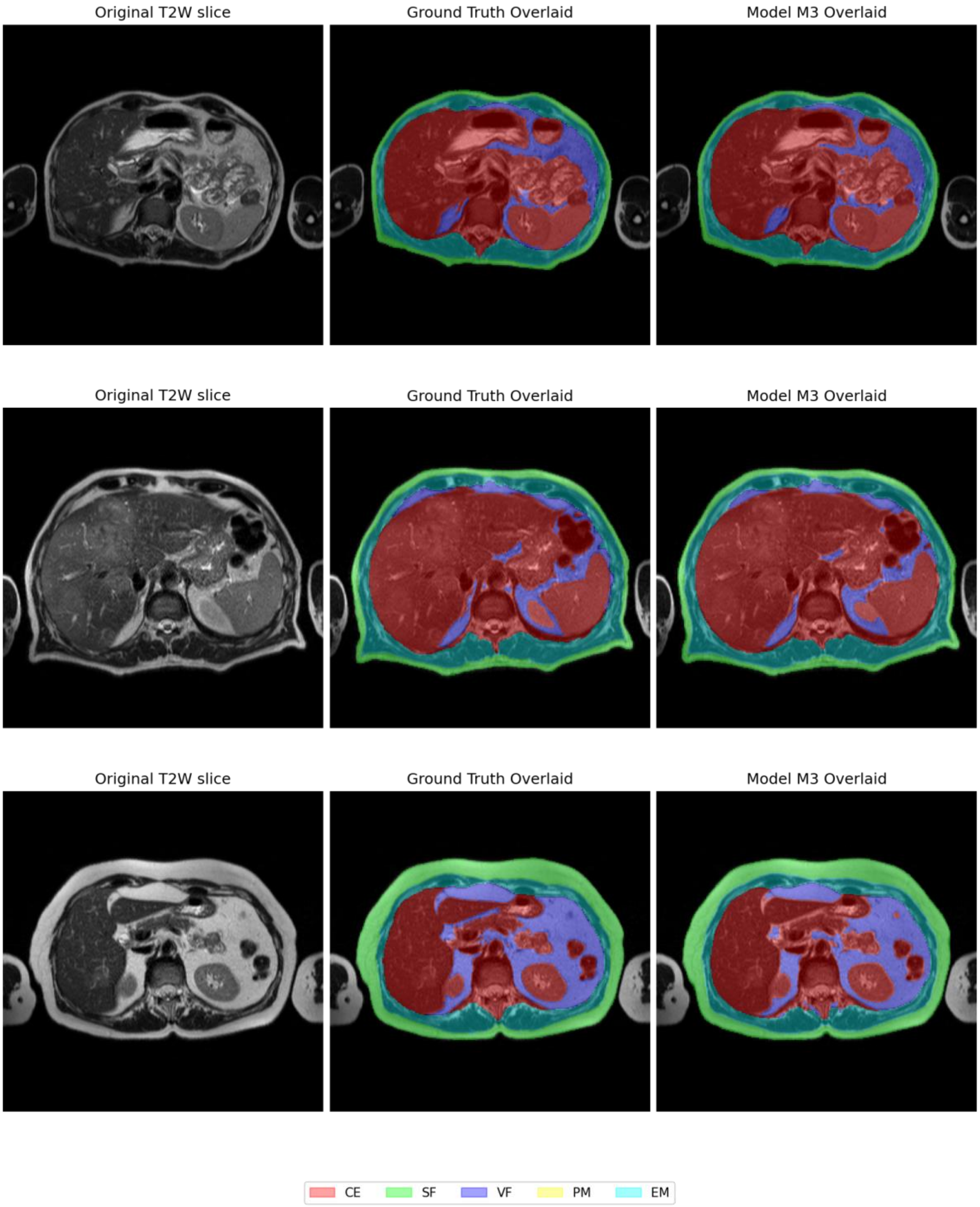

Supplementary Figure 11 : Segmentation results from multiple test patients – sample 3. Each row displays an axial slice from T2W MRI (left), STAPLE-derived ground truth segmentation overlay (middle) and automated segmentation results from the selected DL model M3 (right).

ST1 : RC Limits of Agreement (asymmetric) for tissue volumes computed by different DL models

Model	SF (RC_L)	SF (RC_U)	VF (RC_L)	VF (RC_U)	PM (RC_L)	PM (RC_U)	EM (RC_L)	EM (RC_U)
M1	-10.6	11.9	-24.4	32.3	-49.5	98.0	-4.4	4.6
M2	-10.7	11.9	-23.6	30.9	-46.3	86.1	-5.2	5.5
**M3**	**-10.7**	**12.0**	**-22.6**	**29.1**	**-48.4**	**93.9**	**-5.2**	**5.5**
M4	-10.9	12.2	-23.4	30.5	-45.5	83.5	-5.1	5.4
M5	-10.7	12.0	-24.2	31.9	-47.6	90.8	-4.7	4.9
M6	-10.7	11.9	-23.6	31.0	-48.9	95.7	-4.6	4.8
M7	-10.8	12.1	-24.1	31.8	-47.0	88.5	-4.7	5.0
M8	-10.7	12.0	-23.4	30.6	-46.8	88.0	-5.1	5.4
M9	-10.8	12.1	-23.9	31.5	-45.8	84.6	-5.2	5.5
M10	-10.8	12.1	-23.4	30.6	-45.9	85.0	-5.1	5.3
M11	-10.7	12.0	-23.7	31.0	-47.2	89.3	-4.9	5.2
M12	-10.8	12.1	-24.0	31.5	-46.3	86.2	-5.0	5.3
M13	-10.8	12.1	-24.0	31.6	-46.9	88.2	-5.0	5.3
M14	-10.8	12.1	-23.6	31.0	-46.1	85.4	-5.1	5.4
M15	-10.8	12.1	-23.9	31.3	-46.5	86.9	-5.0	5.3
M16	-10.7	12.0	-33.6	50.5	-49.4	97.6	-5.3	5.6
M17	-10.7	11.9	-33.4	50.0	-47.1	89.2	-5.2	5.5
M18	-10.2	11.4	-29.4	41.7	-49.3	97.4	-5.0	5.3
M19	-10.2	11.4	-29.1	41.0	-48.9	95.5	-5.0	5.2

ST2. Statistical Tests: Paired t-test for tissue volumes at baseline and day 180

**Tissue class**	**T-statistic**	**p-value**
SF	-0.5539	0.5815
VF	-0.2807	0.7798
EM	0.3682	0.7138

Model configuration : CNN-based models

This section outlines the design details of the four U-Net configurations using nnU-Net framework. For a summarised version capturing all major details, see Supplementary Table ST3.

2d

All five folds in the training scheme of 2D configuration accepted 2D image patches of size 192x256 and batch size was set as 35. The images were normalised to a resolution 1.4648 x 1.4648. The U-Net encoder had 6 stages with 2 convolution layers of kernel size [3,3] per stage. The number of features across the stages varied from 32 to a maximum of 520. The decoder had 5 stages with 2 convolution layers of kernel size [3,3] per stage.

3d_fullres

In the training of high-resolution 3D ('3d_fullres') configuration all the five folds accepted 3D image patches of size 24x160x256 and batch size was set as 2. z-score normalisation scheme was used, and the images were normalised to a resolution 4 x 1.4648 x 1.4648. The U-Net encoder had 6 stages with 2 convolution layers of kernel size [3,3,3] per stage. The number of features across the stages varied from 32 to a maximum of 320. The decoder had 5 stages with 2 convolution layers of kernel size [3,3,3] per stage.

3d_lowres

In the training of low-resolution 3D ('3d_lowres') configuration all the five folds accepted 3D image patches of size 24x80x128 and batch size was set as 2. z-score normalisation scheme was used, and the images were normalised to a resolution 2 x 2 x 2. The U-Net encoder had 5 stages with 2 convolution layers of kernel size [3,3,3] per stage. The number of features across the stages varied from 32 to a maximum of 320. The decoder had 4 stages with 2 convolution layers of kernel size [3,3,3] per stage.

3d_cascade

(A cascade configuration where first a 3D U-Net operates on low resolution images and then a second 3D U-Net refines those predictions at higher resolution): This configuration was trained in two stages by cascading the 3d_lowres and 3d_fullres configurations respectively. In this set up, the parameters were inherited from those two individual configurations.

ST3: nnU-Net configurations

	**2d**	**3d_fullres**	**3d_lowres**
**Description**	2D U-Net configuration that operates on patches of 2D slices from the volume	3D U-Net configuration that operates on 3D patches from the volume at high resolution	3D U-Net configuration that operates on 3D patches from the volume at lower resolution
**Target Spacing**	[1.46484375, 1.46484375]	[4.0, 1.46484375, 1.46484375]	[2.0, 2.0, 2.0]
**Number of encoder stages**	6	6	5
**Number of decoder stages**	5	5	4
**Patch size:**	[192, 256]	[48, 160, 256]	[24, 80, 128]
**Batch size:**	35	2	2
**Down sampling strides:**	[[1,1], [2,2], [2,2], [2,2], [2,2], [2,2]]	[[1,1,1], [1,2,2], [2,2,2], [2,2,2], [2,2,2], [1,2,2]]	[[1,1,1], [1,2,2], [2,2,2], [2,2,2], [1,2,2]]
**Convolution kernel sizes:**	[[3,3], [3,3], [3,3], [3,3], [3,3], [3,3]]	[[1,3,3], [3,3,3], [3,3,3], [3,3,3], [3,3,3], [3,3,3]]	[[1,3,3], [3,3,3], [3,3,3], [3,3,3], [3,3,3]]

Model configuration: Transformer-based models

During training, the transformer-based models were optimised to ensure that the models capture sufficient anatomical detail. We experimented by varying the size of input 3D blocks and decoder network features and selected the final model composition based on better validation mean dice scores. The results from experiments on fold 0 are shown in the supplementary figures 12 (UNETR) and 13 (swin-UNETR). Other model configuration parameters used in the final implementation are given below:

- Dimension of hidden layers in the transformer encoder (hidden_size) = 768
- Dimension of the multi-layer perceptrons in the transformer encoder (mlp_dim) = 3072
- Number of attention heads in the multi-headed self-attention block (num_heads) = 12
- Position embedding layer type (pos_embed) = “perceptron”
- Feature normalization type (norm_name) = “instance”
- Boolean argument to determine if residual blocks are used (res_block) = True

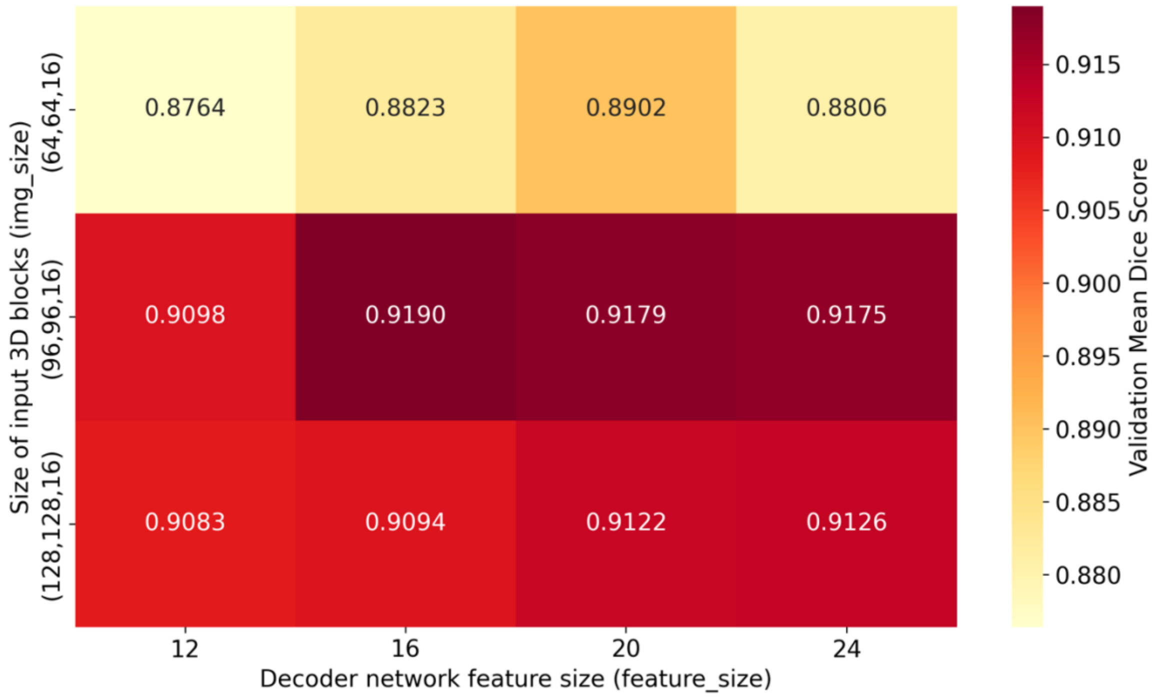

Supplementary Figure 12: Validation Mean Dice Scores for UNETR models from experiments on fold 0. Different combinations were tested by varying the size of input 3D blocks (img_size) and decoder network features (feature_size). The parameters (img_size=96x96x16, feature_size = 16) leading to better validation mean dice scores were selected for final model configuration.

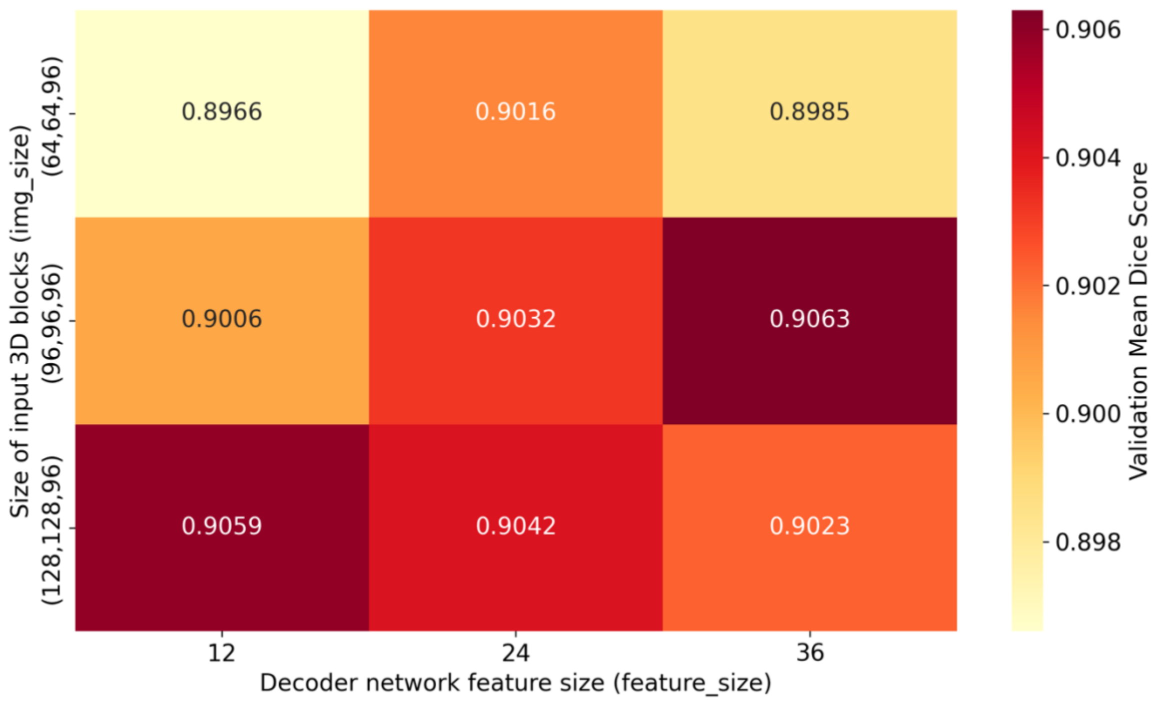

Supplementary Figure 13: Validation Mean Dice Scores for swin-UNETR models from experiments on fold 0. Different combinations were tested by varying the size of input 3D blocks (img_size) and decoder network features (feature_size). The parameters (img_size=96x96x96, feature_size = 36) leading to better validation mean dice scores were selected for final model configuration.

Glossary

nnU-Net: A self-configuring deep learning method for medical image segmentation with U-Net formations capable of adapting automatically to new datasets.
UNETR: UNETR (UNet TRansformers) is a hybrid architecture designed for volumetric medical image segmentation tasks with a Vision Transformer as the encoder.
Swin-UNETR: A medical image segmentation model that combines the Swin Transformer architecture with the UNETR.
FOV: Field Of View
DSC: Dice Similarity Coefficient.
wCV: within-subject Coefficient of Variation.
ICC: Intra-class Correlation Coefficient.
LOA: Limits Of Agreement.
Training: The process of teaching a machine learning model using labelled data.
Validation: A subset of data used to evaluate model performance during training and tune hyperparameters.
Testing: The final evaluation of a trained model using a separate, unseen dataset.
Held-out test set: A portion of data completely separated from training and validation, used for final model evaluation.
Fold: One of the subsets of data used in cross-validation for training and validation.
Data augmentation: A technique to artificially increase the size and diversity of a training dataset by applying various transformations to existing data.
Hyperparameters: Configuration settings for machine learning algorithms that are set before training and affect model performance.
Epoch: One complete pass through the entire training dataset during model training.
Iteration: A single update of the model's parameters using a batch of data.
Ensemble: A technique that combines multiple models to improve overall performance and robustness.
